# Myocarditis successfully diagnosed and controlled with speckle tracking echocardiography

**DOI:** 10.1186/s12947-020-00203-4

**Published:** 2020-06-12

**Authors:** Beata Uziębło-Życzkowska, Marta Mielniczuk, Robert Ryczek, Paweł Krzesiński

**Affiliations:** Department of Cardiology and Internal Diseases, Military Institute of Medicine, Szaserów Street 128, 04-141 Warsaw, Poland

**Keywords:** Cardiac magnetic resonance, Myocarditis, Speckle tracking echocardiography

## Abstract

**Background:**

Speckle tracking echocardiography (STE) is an objective, well-validated and reproducible technique of assessing left ventricular longitudinal deformation; it also offers a more sensitive assessment of myocardial contractility than widely used visual estimation. Evaluating global longitudinal strain may help identify patients with subclinical left ventricular systolic dysfunction.

**Case presentation:**

We report the case of a 28-year-old man with myocarditis, which successfully diagnosed and followed-up with the novel echocardiography method using speckle tracking imaging. The patient was referred to our hospital with an initial diagnosis of ST-segment elevation myocardial infarction. Transthoracic echocardiography did not show any contractility abnormalities. Hence, in the course of further diagnostics, left ventricular function was assessed by STE. Depressed global longitudinal strain was noted within several segments of the left ventricle. Then, cardiac magnetic resonance imaging was performed to confirm the diagnosis of myocarditis.

**Conclusions:**

STE seems to be competitive in relation to cardiac magnetic resonance imaging in the diagnosis of some diseases, such as myocarditis.

## Background

Patients with acute myocarditis may present various clinical scenarios that often mimic other cardiac abnormalities. The clinical manifestations of myocarditis can extensively vary, ranging from completely asymptomatic to acute left ventricular (LV) failure leading to sudden cardiac death [1, 2]. Both acute and fulminant myocarditis, as well as chronic ones, can lead to advanced heart failure, so early diagnosis of this disease is important. Due to the wide spectrum of clinical presentations and symptoms reported by patients, early diagnosis of myocarditis is very rare [3]. The reported signs are mostly unspecific, such as dyspnoea (72%), chest pain (32%) and arrhythmias (18%) [4], and relate to more common cardiovascular diseases. Both genetic and environmental factors can lead to myocardial injury, and their influence is usually intertwined [1]. One of the myocarditis triggers can be viral infection. For this reason, symptoms of viral infection, such as fever, cough and myalgia, often coexist or precede myocarditis. On the other hand, studies show that the course of myocarditis during a viral infection can be completely asymptomatic and may even be undiagnosed by non-invasive cardiac imaging [5].

Choosing the optimal diagnostic method is also difficult because the amount of myocardial damage varies widely. If the affected area is small, standard echocardiography may not be sensitive enough to detect the typical findings that would allow for proper diagnosis. Cardiac magnetic resonance (CMR) is the diagnostic modality of choice in such cases, as it shows small inflamed myocardial areas. Speckle tracking echocardiography (STE) has been proven to be a valuable method in detecting subclinical contractility abnormalities in various clinical situations. Thus, it may also be helpful in managing patients with myocarditis.

## Case report

We present the case of a 28-year-old man admitted to the Cardiology Department due to suspected ST-elevation myocardial infarction (STEMI). He is an active smoker with no other cardiovascular risk factors and no previous history of cardiovascular diseases. Several days before admission, he was treated for sore throat. The previous day, he felt chest pain radiating to his left shoulder. Physical examination at admission did not reveal any significant findings. The electrocardiogram showed a sinus rhythm of 75 bpm and a concave ST segment elevation in leads II, III, aVF and V5–V6. The initial laboratory tests revealed increased levels of high-sensitivity troponin T at 901 ng/L [normal level < 14 ng/L], N-terminal pro-B-type natriuretic peptide at 612 pg/ml and increased inflammatory markers: white blood cells at 21.8 × 10^9^ /l [normal range 4.0–10.0], erythrocyte sedimentation rate at 58 mm [0–8] and C-reactive protein at 15.2 mg/dl [0–0.8]. Transthoracic echocardiography (GE Vivid E95) did not show any contractility disorders (left ventricular ejection fraction - LVEF - 61%). However, due to suspected STEMI, the patient underwent coronarography, which showed no stenosis in the coronary arteries.

**Fig. 1:**
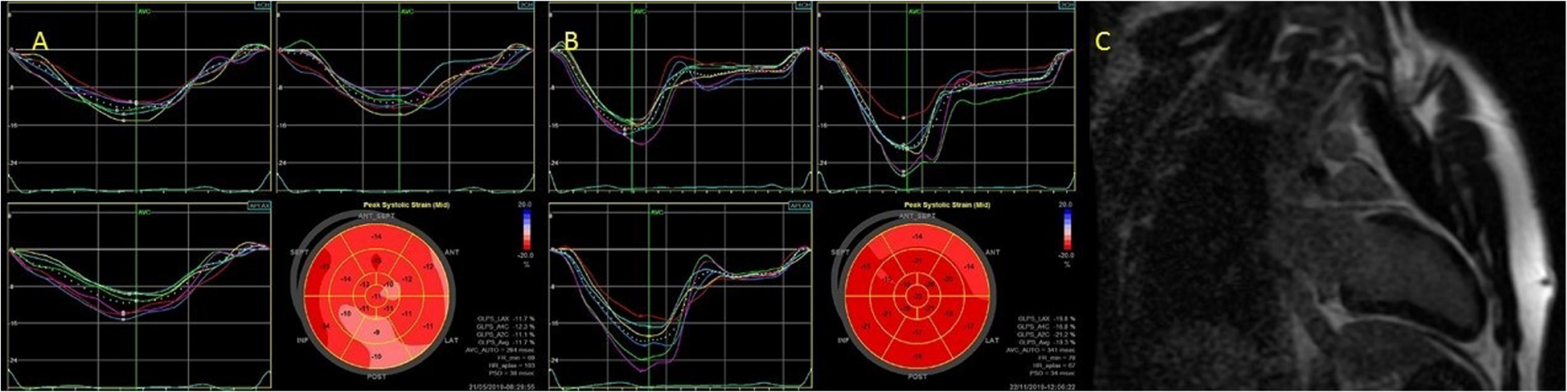
**a** - The decreased global longitudinal strain (GLS) -11.7% on the second day of hospitalization, the lowest GLS values were found within the basal and middle segments of the posterior (− 10% and − 9% respectively), lateral (− 11% and − 11%) and anterior (− 12% and − 12%) walls; **b** – Improvement in the left ventricle contractility measured by GLS after 3 months of treatment. **c** - In the images of late post-contrast enhancement visible discrete, subepicardial areas of post-contrast reinforcement within the basal segments of the inferior, infero-lateral and infero-septal wall

In the course of further diagnostics, LV performance was assessed by STE. Depressed global longitudinal strain (GLS) was noted at − 11.7% [normal range < − 20%], with the lowest values within the basal and the middle segments of the posterior (− 10% and − 9%, respectively), lateral (− 11% and − 11%) and anterior (− 12% and − 12%) walls (Fig. 1a). CMR was performed 10 days after the onset of symptoms to confirm the suspicion of myocarditis. In the late gadolinium enhancement (LGE) sequences, discrete and sub-epicardial areas of post-contrast reinforcement were shown within the basal segments of the inferior, infero-lateral and infero-septal LV walls (Fig. 1c). The LV size and LVEF (61%) were normal. The localisations of area of myocarditis showed on CMR conclusions corresponded to area with depressed longitudinal strain that of STE on echo. To prevent developing LV systolic dysfunction, angiotensin-converting enzyme inhibitor and beta-blocker were applied.

Six months later, on the follow-up visit, STE showed significant improvement in LV deformation, both regional and global (Fig. 1b).

Currently, the patient is in good general condition. He has returned to work and began to undertake regular physical activity.

## Discussion

Acute coronary syndrome (ACS) appears to be one of the more common clinical manifestations of myocarditis. Myocarditis should be especially kept in mind when treating young people with symptoms suggestive of ACS but with normal coronary angiograms. Differential diagnosis is particularly difficult due to similar clinical symptoms and laboratory test results. Typical anginal chest pain and electrocardiogram patterns, as well as increased level of cardiac enzymes, are common symptoms for both of these states. Moreover, echocardiography findings can also be similar. Angelini et al. evaluated 12 patients with normal coronary arteries admitted to the hospital due to suspected ACS and who underwent endomyocardial biopsy (EMB) [6]. Based on the Dallas criteria, they diagnosed myocarditis in seven of the 12 patients. Using other immunohistochemical and molecular methods, the authors detected the attributes of myocarditis in four other patients. Instances of myocarditis mimicking ACS have also been described by other authors [7]. The challenge remains in choosing the appropriate method for diagnosing myocarditis in such patients, especially since the Dallas criteria were proven to be not that sensitive in a large study [8].

Differential diagnosing of myocarditis may be difficult; establishing its aetiology and detecting systolic LV dysfunction may also be challenging sometimes demands using sophisticated diagnostic modalities. EMB remains the diagnostic gold standard, but its invasive nature with risk of complications, causes that is not widely used and is limited to specific indications [9]. Moreover, as we mentioned above, even EMB, an invasive procedure with risk of complications does not recognise all cases of myocarditis.

CMR seems to be the most accurate non-invasive diagnostic modality in myocarditis, but its availability is still limited. In the paper of the International Consensus Group “Cardiovascular Magnetic Resonance in Myocarditis”, terminologies for describing CMR findings in myocarditis [10] have been proposed. According to this document, myocardial oedema, increased myocardial early gadolinium enhancement ratio and regional LGE are CMR findings that are consistent with myocardial inflammation. However, it is strongly emphasised that one non-invasive imaging method is usually not sufficient to confirm the diagnosis of myocarditis.

Conventional echocardiography is usually the first procedure performed in the case of suspected cardiac dysfunction. Felker et al. presented that standard echocardiography parameters measured during the early time of hospitalisation can play an important role in classifying a patient with myocarditis [11]. They revealed that patients with fulminant myocarditis present with nondilated, thickened and hypocontractile LV. By contrast, patients with acute myocarditis are characterised by LV dilatation, normal LV thickness and decreased LV systolic function. However, standard echocardiography has several limitations in the evaluation of LV performance in myocarditis, especially in patients with preserved LVEF. In such cases, GLS, is more promising. This novel technique of assessing myocardial efficiency seems to be a sensitive measure of LV contractility [12–14], detecting subtle but clinically relevant LV dysfunction that cannot be captured by standard visual estimation of contractility. There is growing evidence that discrete impairment of LV performance measured by STE may be considered a diagnostic tool; it may also be of prognostic significance in many cardiac and non-cardiac conditions [13–15]. Moreover, a recent study revealed good inter-technique agreement in strain measurements between STE and CMR techniques [16]. The results of this study also confirmed that myocardial strain derived by both STE and CMR techniques is highly reproducible. The usefulness of the regional LV systolic dysfunction assessed by STE was shown in the study of Leitman et al. [17]. This study revealed a high correlation between the regional strain values and the presence of delayed enhancement in the same LV segments in patients with myocarditis/perimyocarditis/myopericarditis. The authors also pointed out that in individuals considered to be afflicted with inflammatory heart diseases, the locations of the regional wall motion abnormalities are similar for both STE and CMR, and were mainly focused in the lateral, inferior and posterior wall segments. These findings confirmed the high correlation between global strain assessed by both STE and CMR. Similar findings were described by Kostakou et al. [18]. This study demonstrated that patients with acute myocarditis, similar to the presented case, had significantly impaired global LV GLS despite having normal LVEF. Additionally, the results of the regional LV systolic dysfunction assessed by STE corresponded to the LGE changes in the same LV segments. The authors hypothesised that STE could be a promising method for the detection of LV regional fibrosis. They pointed out that impairment of the GLS of the lateral segments LV has an especially great sensitivity and specificity in diagnosing myocarditis. It also turned out that two-dimensional STE showed high correlation with the amount and localisation of the oedema found in CMR in patients with acute myocarditis [19]. In chronic myocarditis with preserved LVEF, the LV GLS was also impaired compared to the control subjects [20].

In our patient, similar to the studies mentioned above, the global LV GLS was impaired at the onset of the symptoms. The locations of the regional LV strain abnormalities were comparable to those described in CMR. Moreover, the locations of the segments with impaired GLS in our patient were similar to those that are characteristic of inflammatory heart diseases [17]. These regional and global LV strain values improved and normalised along with clinical improvement. Finally, it is worth mentioning that echocardiography can be used not only in the diagnosis and control of myocarditis treatment, but also to predict adverse outcomes in myocarditis [21].

In summary, the high reproducibility implies that strain techniques may be particularly valuable, not only in diagnosis, but also in the follow-up of the course of a patient’s disease. This finding is very important from a clinical perspective, especially in patients with preserved LVEF and suspected myocarditis.

## Conclusions

The presented case proves that GLS provides correct diagnosis, justifies the implementation of pharmacotherapy and enables a simple assessment of the treatment effects in myocarditis.

## Data Availability

The data of this study are available from the corresponding author upon request.

## Abbreviations

ACS: Acute coronary syndrome
CMR: Cardiac magnetic resonance
EMB: Endomyocardial biopsy
GLS: Global longitudinal strain
LGE: Late gadolinium enhancement
LV: Left ventricular
LVEF: Left ventricular ejection fraction
STE: Speckle tracking echocardiography
STEMI: ST-elevation myocardial infarction
